# Effect of Aging Time on Meat Quality of Longissimus Dorsi from Yunling Cattle: A New Hybrid Beef Cattle

**DOI:** 10.3390/ani10101897

**Published:** 2020-10-16

**Authors:** Yongliang Fan, Ziyin Han, Abdelaziz Adam Idriss ARBAB, Yi Yang, Zhangping Yang

**Affiliations:** 1 College of Animal Science and Technology, Yangzhou University, Yangzhou 225009, China; dx120170088@yzu.edu.cn (Y.F.); ZiyinHan@126.com (Z.H.); arbabtor@yahoo.com (A.A.I.A.); 2 Joint International Research Laboratory of Agriculture & Agri-Product Safety, Ministry of Education, Yangzhou University, Yangzhou 225009, China; 3 Jiangsu Co-Innovation Center for the Prevention and Control of Important Animal Infectious Diseases and Zoonoses, College of Veterinary Medicine, Yangzhou University, Yangzhou 225009, China; yangyi@yzu.edu.cn

**Keywords:** Yunling cattle, aging time, meat quality, growth performance, carcass traits

## Abstract

**Simple Summary:**

Beef is in great demand in many countries. Consumers are more inclined to buy beef with good tenderness, color, flavor, and healthy fatty acid composition. Beef that has been aged is often more suitable for cooking and processing. A research gap exists regarding the analysis of meat quality during the aging process in Yunling cattle, a new hybrid beef cattle bred by Chinese researchers. This study measured a set of indicators of Yunling beef and other two breeds as controls (Simmental cattle, an excellent beef cattle breed used around the world, and Wenshan cattle, a local beef cattle breed in southern China), including the pH, water loss rate, cooking loss, Warner-Bratzler shear force, myofibrillar fragmentation index, inosine 5′-monophosphate, color, and fatty acid profiles. In addition, some growth performance and carcass characteristics were determined. The results showed that the pH, water holding capacity, growth performance, and carcass traits of Yunling and Simmental cattle were basically the same and better, respectively, than those of Wenshan cattle. Aging time had no effect on beef fatty acid composition, with Yunling beef showing a healthier fatty acid profile versus the other two breeds. With increased aging time, the tenderness and color of Yunling beef became more suitable for cooking and sensory characteristics. Therefore, this study suggests that Yunling beef should be used for cooking and processing after aging.

**Abstract:**

The beef aging process is essential for compliance with certain major requisites, such as sensory characteristics for cooking and meat processing. Meat quality analysis of Yunling cattle, a new hybrid beef cattle bred by Chinese researchers, during the aging process, represents a major research gap. To explore Yunling beef initially, indicators associated with meat quality during the aging process of Yunling, Simmental, and Wenshan cattle were measured. In addition, some important economic traits were detected in the three breeds, including growth performance and carcass characteristics. The results showed that the growth performance, carcass traits, pH, and water holding capacity of Yunling and Simmental cattle were basically the same and better, respectively, than those of Wenshan cattle. The proportions of individual fatty acids in Yunling beef were healthier than in the other two breeds. Aging time did not affect the fatty acid profiles of the beef (*p* > 0.05). The contents of certain fatty acids in the three beef types displayed some differences in terms of days of aging (*p* < 0.05). The tenderness and meat color were better in the Yunling beef as the aging time increased, indicating that Yunling beef aged for 7 days was more suitable for cooking, exhibiting better sensory characteristics. Thus, a 7-day short-term aging process is very effective in improving the quality of Yunling beef. Our study attempted to fill a gap in the Yunling beef quality analysis during aging, providing further evidence for Yunling beef improvement.

## 1. Introduction

Beef is a frequently consumed source of red meat, with high demand for the product worldwide [1,2,3]. Beef is an excellent source of protein, essential vitamins, and minerals in the human diet [4]. Chinese native cattle are better adapted to the environment versus introduced varieties. Yunling cattle, which are attracting increasing attention, was the fourth beef cattle variety with completely independent intellectual property rights developed by Chinese scientific researchers. Yunling cattle represent not only the first meat cattle breed bred by three-way hybridization in China, but were also the first new beef cattle breed adapted to the tropical and subtropical regions of southern China. Its ultimate genetic composition is 1/2 Brahmin, 1/4 Murray Grey, and 1/4 Yunnan Yellow cattle [5]. Yunling cattle possess antiparasitic and high production potential advantages, and are also capable of adapting to tropical and subtropical environments. However, the limited research that currently exists on Yunling cattle focuses only on molecular breeding [5,6]. The effect of aging time on the quality of Yunling beef is still unknown.

Economic return in beef cattle rearing is affected by daily weight gain (ADG) [7]. Body weight (BW), carcass weight, and dressing percentage comprise the carcass traits, which are important in meat production [8]. Good economic performance alone is not enough. Essentially, consumer perception of meat quality is a vital standard for meat selection [9]. Consumers in China commonly think that fresh meat is more edible than meat that has been aged [10]. However, the aging progress is essential for beef to comply with certain major requisites, such as sensory characteristics for cooking and meat processing [11,12]. In the European Union (EU), only aged beef is allowed to be sold. Beef was previously reported to be able to be sold in France after being aged for at least 6–8 days [13]. A collection of compounds is produced in meat to enhance meat flavor during aging. These flavor compounds are mostly amino acids arising from the proteolysis of muscle cellular enzymes rather than microbial decomposition [14]. Postmortem aging is further necessary to improve beef tenderness [15]. Additionally, a family of indicators related to meat quality showed dynamic changes related to the aging process, such as changes in pH, water loss rate, Warner-Bratzler shear force (WBSF), inosine monophosphate (IMP), and meat color. Therefore, the need exists for exploration of the effect of aging time.

To fill the gap of the Yunling beef quality analysis during aging, this study analyzed the chemical composition, physicochemical properties and fatty acid profiles of Yunling beef. In addition, differences were compared among three breeds (Yunling, Simmental, and Wenshan cattle). Our findings provide further evidence for Yunling beef improvement.

## 2. Materials and Methods

### 2.1. Ethics Statement

The feeding and slaughter experiment were conducted at the Grassland Animal Science Research Institute of Yunnan province (25°02′ N and 102°43′ E). The carcass characteristics and meat quality were analyzed at the College of Animal Science and Technology, Yangzhou University, Yangzhou, Jiangsu province, China.

All experimental animals were performed in strict accordance with animal management regulations (The People’s Republic of China Ministry of Science and Technology, revised in 2004) and approved by the Institutional Animal Care and Use Committee (IACUC) of the Yangzhou University Animal Experiments Ethics Committee (Permit Number: SYXK (Su) IACUC 2012-0029). All efforts were made to minimize the suffering of the cattle.

### 2.2. Animals and Management

In this study, 15 11-month-old cattle were selected from each breed (Yunling, Simmental, and Wenshan cattle) and placed in an independent closed-ended barn for physical examination, parasite expulsion, and routine epidemic prevention. The barn was 12 m wide, 40 m long, and 3.5 m high, with a maximum capacity of 60 cattle and a playground of about 600 square meters facing the sun. The cattle were pre-fed for 1 month to eliminate dietary stress [16]. The diet consisted of a 35:65 (DM %) concentration ratio of dry matter (Table S1). The cattle were fed twice a day with adequate water and manure was cleaned up daily. The trial period was 180 days, which was consistent with the feeding conditions in the pre-feeding period.

### 2.3. Growth Performance and Carcass Traits

The fattening period lasted for 6 months, starting at the twelfth month of age and finishing at the eighteenth month of age. The average fasting weights of the first 2 days and the last 2 days were, respectively, the initial and final weights during the fattening period. At the end of the trial period, the ADGs of each breed were calculated using the following formula: ADG = (final weight − initial weight)/180. Movement in the playground and feed intake were prohibited for 24 and 12 h, respectively, prior to slaughter. After slaughter, the BW of 15 cattle were recorded. To obtain the carcasses, the 15 cattle were stunned via electric shock and bled, followed by removal of their heads, hooves, and tails. Finally, they were skinned and the viscera were removed. The carcasses were divided into left and right halves along the middle of the spine with a chainsaw and dressed, thereby obtaining the half carcasses. The half carcasses were cut into two quarter carcasses between the twelfth and thirteenth ribs at the waist, thereby obtaining the quarter carcasses [17,18]. The carcass weights were measured after the quarter carcasses were stored at 4 °C for 24 h. The slaughter rate was calculated using the following formula: dressing percentage (%) = carcass weight/BW × 100.

### 2.4. Chemical Composition

Approximately 350 g of longissimus dorsi (LD) samples between the twelfth to thirteenth ribs of each carcass were taken and aged for 7 days under vacuum conditions at 4 °C to evaluate meat quality [19]. Sampling was performed on days 0, 1, 2, 3, 5, and 7 [20].

Beef composition was measured using a Foss Lab Meat/Food Composition fast analyzer (FOSS Ltd., Hillerød, Denmark). Samples weighing 200 g on days 0, 3, and 7 were separated from the LD sample and extended into the analysis plate. The results were the averages of 16 technical replicates per sample. The equipment was calibrated according to the Soxhlet method [21]. The protein, collagen, moisture, and fat contents were recorded.

### 2.5. pH and Water Loss Rate

The pH values of each sample collected on days 0, 1, 2, 3, 5, and 7 were measured with a pH meter (Eutech Instruments, pH Spear, Massachusetts, MA, USA) [22]. Calibration of the pH electrode was performed with standardized buffers (pH 4.0 and 7.0). Each LD sample was measured three times.

Pieces of meat measuring 2 × 2 × 1 cm were cut from the LD samples collected on days 0, 1, 2, 3, 5, and 7. Next, a pressure of 343 N (35 kg) was applied and maintained for 5 min. The water loss rate was calculated according to the following formula:

water loss rate (%) = (W_w1_ − W_w2_)/W_w1_ × 100%, (1)

where W_w1_ represents the weight of the sample before water loss and W_w2_ represents the weight of the sample after water loss. Each LD sample was measured three times.

### 2.6. Cooking Loss and Shear Force

The cooking loss rate was measured using the direct weight method [23]. Samples of LD weighing 30 g were taken from the LD samples collected on days 0, 1, 2, 3, 5, and 7, then wrapped and sealed in a cooking bag and cooked in a water bath at 80 °C. When the central temperature reached 70 °C, the samples were unwrapped and the surface moisture was dried. The cooking loss rate was calculated according to the formula:

cooking loss (%) = (Wc_1_ − Wc_2_)/Wc_1_ × 100% (2)

where Wc_1_ represents the weight of the sample before cooking and Wc_2_ represents the weight of the sample after cooking. Next, to measure the WBSF, approximately 3 cm of LD sample was removed from the cooked LD sample, and the WBSF was measured using the method set out by Luo et al. [24]. Each LD sample was measured three times.

### 2.7. MFI and IMP Assay

LD samples weighing 10 g each taken from the LD samples were frozen and stored at −80 °C until further analysis of inosine 5′-monophosphate (IMP). The myofibrillar fragmentation index (MFI) of the LD samples collected at days 0, 3, and 7 was determined using the method described by Culler et al. [25] and Hopkins et al. [26]. LD samples weighing 4 g each were taken from the fat connective tissue, weighed, and transferred to a 50 mL polypropylene tube. The samples were homogenized with an ultrasonic cell crusher at 4 °C after adding 40 mL MFI extract (100 mmol/L KCl, 7 mmol/L KH_2_PO_4_, 18 mmol/L K_2_HPO_4_, 1 mmol/L EDTA, 1 mmol/L MgCl_2_, pH = 7.0). The homogenized samples were centrifuged at 10,000 r/min for 15 min and the supernatant was removed. The deposits were extracted again according to the abovementioned conditions. After dissolving the final deposit with 15 mL MFI extract, the myofibrillar extract was obtained by filtering the solution with a single layer gauze to remove tissue and cellular debris. Protein concentrations of the myofibrillar extract were determined using the biuret method, and the MFI extract was used to adjust that protein concentration to 0.5 mg/mL in the myofibrillar extract. Absorbance of the extract was measured at a wavelength of 540 nm and each sample was detected three times. MFI was calculated using the formula A540 nm × 200.

Inosine monophosphate (IMP) assay was performed according to the method described by Tikk et al. [27]. LD samples weighing 10 g each were thawed and 800 mg of LD separated from the LD samples on days 0, 3, and 7 were transferred to a 50 mL polypropylene tube, where 3 mL (7%) of perchloric acid (CPA) was added. After centrifuging for 15 min at 15,000 rpm, the supernatant was transferred to another 50 mL polypropylene tube. Then, l.44 mL of 0.85 M K_2_CO_3_ was added to the polypropylene tube and the mixture was centrifuged once more. Afterwards, the supernatant was syringe filtered (0.22 μm filter), and 10 μL of the supernatant was injected into the HPLC system (Agilent1100). Analytes were detected using a UV detector at a wavelength of 254 nm. The results were analyzed using the external standard method and the analytes were quantitated based on retention time and peak area of the analyte.

### 2.8. Color Measurement

Samples collected at days 0, 1, 2, 3, 5, and 7 were exposed in air for 30 min (blooming) at 4 °C to measure color, using a Chroma Meter CR-400 colorimeter (Konica Minolta, Inc., Tokyo, Japan) based on luminance (L*), redness (a*), and yellowness (b*) in the CIELab color space. The color values were the averages of six scans for each LD. The C* and H* values were calculated from the a* and b* values using the following respective formulae [28,29]:

C* = (a*^2^ + b*^2^)^0.5^ and H* = arctan (b*/a*) (3)

### 2.9. Fatty Acid Profile Detection

To analyze the fatty acid profiles, frozen LD samples collected at days 0, 3, and 7 were crushed with a mortar and dried at 103 °C for 1 h in an oven. Two grams of the dried powder sample were weighed and transferred into a 10 mL polypropylene centrifuge tube containing chloroform-methanol (2:1, *v*/*v*). Then, the mixture was vortexed for 10 s and kept at room temperature for 48 h. After filtering, 4 mL of 20% NaOH was added to the filtered extract. After vortexing for 10 s, 5 mL of chloroform-methanol (2:1, *v*/*v*) was added to the mixture. After vortexing for 10 s, the final mixture was kept at room temperature until layering. Afterward, 2 mL of the lower liquid was transferred to a test tube, and 1 mL of anhydrous ethanol was added. After vortexing, 1 mL of ether was added, and the mixture was vortexed again. Then, 1 mL of NaOH-methanol (2:1, *v*/*v*) was added. The test tube was then placed in a water bath at 100 °C for 10 min, which was followed by vortexing. After adding 2 mL of boron trifluoride, the mixture was homogenized using a rotating vibration blender and placed in a water bath (100 °C) for 1 min. Next, 2 mL of hexyl hydride was added and homogenized, 3 mL of sodium chloride was added, and then the mixture was homogenized. After centrifugation at 900× *g* for 5 min, 1.5 mL of supernatant was transferred to a 2 mL brown vial and used for GC analysis. Separation was carried out using a capillary column HP-88 with the following temperature program: 55 °C for 3 min, then increased by 13 °C/min to 175 °C, then increased by 4 °C/min to 210 °C, where the temperature was held for 20 min. The injector temperature was 270 °C and the detector temperature was 280 °C.

### 2.10. Statistical Analysis

Statistical analysis was performed by two-way Analysis of Variance (ANOVA) using Tukey-Kramer adjusted generalized linear model (GLM) procedures of Statistical Analysis Software (SAS) 9.4 (SAS Institute, Cary, NC, USA). Differences were considered significant at the *p* < 0.05 level.

## 3. Results

### 3.1. Growth Performance and Carcass Traits

The initial weight, final weight, LW, ADG, carcass weight and dressing percentage of the experimental cattle are shown in Table 1. Yunling cattle’s initial and carcass weights were similar to Simmental cattle and significantly higher than Wenshan cattle (*p* < 0.05). The LW of Yunling cattle was significantly lower than in Simmental cattle (*p* < 0.05) and significantly higher than in Wenshan cattle (*p* < 0.05). There were no differences in ADG or dressing percentage between Yunling and Simmental cattle (*p* > 0.05). The dressing percentage of Wenshan cattle was lower (*p* < 0.05) than other tested breeds.

### 3.2. Chemical Composition

The chemical contents of the types of beef are shown in Table 2. The protein, moisture, and fat contents were significantly different among the three breeds (*p* < 0.05), while the collagen content was not different among the three breeds (*p* > 0.05). The protein content of Yunling cattle was higher than the other breeds on the same aging day (*p* < 0.05). The moisture contents of Yunling and Simmental cattle were lower than in Wenshan cattle (*p* < 0.05). The fat contents were not significantly different between Yunling and Simental cattle (*p* < 0.05), but both were significantly higher than in Wenshan cattle (*p* < 0.05). In addition, no significant differences were found for protein, pH, collagen, moisture, or fat among the three breeds at different aging times (*p* > 0.05). No breed × aging time interaction effects were found for protein, collagen, moisture, or fat contents (*p* > 0.05).

### 3.3. pH and Water Loss Rate

The effects of breed and aging time on pH and water loss rate are shown in Table 3. The pH was affected by aging time (*p* < 0.05). The pH of Yunling cattle beef decreased on day 1 compared to day 0, then increased on day 5 compared to day 3 (*p* < 0.05). The pH values of Simmental and Wenshan cattle exhibited sustained and significant decreases from day 0 to day 2 (*p* < 0.05). There were no differences between the three tested breeds (*p* > 0.05), except on days 0 and 3. At day 0, the pH of the Wenshan cattle beef was higher than that of Simmental cattle (*p* < 0.05), and differences in pH were observed between Yunling cattle beef and the other two breeds (*p* > 0.05). At day 3, a higher pH was shown in Simmental cattle beef compared to Yunling and Wenshan cattle beef (*p* < 0.05), respectively.

The water loss rate was not influenced by breeds other than on day 1. The water loss rate of Simmental beef on day 1 was lower than that on day 3 (*p* < 0.05). Aging time significantly affected the water loss rate of Simmental and Wenshan cattle, but not Yunling cattle. The water loss rate of Simmental beef on day 1 was lower than on day 0 (*p* < 0.05) and higher on day 3 than on day 2 (*p* < 0.05). The differences in water loss rate for Wenshan beef increased between day 0 and 1 (*p* < 0.05). A breed × aging time interaction effect was observed for both pH and water loss rate (*p* < 0.05).

### 3.4. Cooking Loss and Warner-Bratzler Shear Force

The cooking loss and WBSF are shown in Table 4. Cooking loss was shown to be significantly influenced by breed and aging time (*p* < 0.05). Interaction effects caused by breed × aging time appeared in cooking loss and WBSF (*p* < 0.05). The cooking loss of Simmental and Wenshan cattle rose and then leveled off. The differences in cooking loss in Yunling cattle appeared at days 2, 3, and 5 of aging time. The cooking loss rates of all breeds at day 7 were higher than day 0 (*p* < 0.05). The WBSF was also significantly influenced by breed and aging time (*p* < 0.05). For Yunling and Wenshan cattle, significant decreases of WBSF occurred at day 5 and 7 (*p* < 0.05). For Simmental cattle, the WBSF decreased significantly on day 7 (*p* < 0.05).

### 3.5. MFI and IMP Assay

The MFI and IMP were significantly affected by breed and aging time (*p* < 0.05) (Table 5). There was no breed × aging time interaction effects in either MFI or IMP (*p* > 0.05). The MFI values of the three breeds significantly rose on days 0, 3 and 7 (*p* < 0.05) and the MFI of Yunling cattle was higher than the values of both Simmental and Wenshan cattle at days 0, 3 and 7 (*p* < 0.05). For Yunling cattle, IMP content declined at day 3 and 7 (*p* < 0.05). For Simmental cattle, IMP content declined at day 3 (*p* < 0.05). For Wenshan cattle, IMP content declined at day 7 (*p* < 0.05).

### 3.6. Color Measurement

Breed affected L* and a* (*p* < 0.05) but did not affect b* (*p* > 0.05) (Table 6). The decrease in L* value among the three breeds cattle only occurred on day 0 (*p* < 0.05). No breed × aging time interaction effects occurred in L*, a* or b* (*p* > 0.05). The decrease in the a* value of the three breeds occurred on days 0, 2, 5, and 7 (*p* < 0.05). The a* values of the Yunling and Simmental cattle were significantly higher than in Wenshan cattle at days 0, 2, and 5 (*p* < 0.05). The a* value of Simmental cattle was significantly higher than in Yunling and Wenshan cattle at day 7 (*p* < 0.05). The L*, a* and b* values were influenced by aging time (Table 6). Higher a* values were observed in the beef types with increasing aging time, leading to a more intense redness of the meat. All three breeds of beef were brighter and yellower as the L* value increased on day 0 compared with day 7.

### 3.7. Fatty Acid Profile Detection

The proportions of individual fatty acids compared to total fatty acids are shown in Table 7, Table 8 and Table 9. The percentages of fatty acids from each beef type showed negligible variation in regard to the aging process (*p* > 0.05). The breed × aging time interaction exerted no effect on any fatty acid (*p* > 0.05). With the exceptions of C17:0, C18:2n6t, C18:3n3, and C22:6n3, the contents of the fatty acids were different between the three breeds (*p* < 0.05). The contents of SFAs were higher in Yunling beef than in Simmental and Wenshan beef at days 0, 3, and 7 (*p* < 0.05). In contrast, the contents of MUFAs were lower in Yunling beef than in Simmental and Wenshan beef at days 0, 3, and 7 (*p* < 0.05). Additionally, PUFA contents were higher in Yunling and Simmental beef than in Wenshan beef at days 0, 3, and 7 (*p* < 0.05), with no differences in the values of P:S among the test breeds (*p* > 0.05). The C16:0 content of the Yunling cattle was significantly higher than the contents of Simmental cattle and Wenshan cattle on all test days (*p* < 0.05). The C18:2n6c content of Yunling cattle was significantly higher than the contents seen in Simmental cattle and Wenshan cattle (*p* < 0.05).

## 4. Discussion

### 4.1. Growth Performance and Carcass Traits Analysis

The ADG reflects the weight gain of cattle per unit of time. ADG is not only an important factor affecting the number of days of the fattening period [7], but also affects muscle and adipose tissue growth [30]. Reynolds et al. [31] found that the ADG could be used as an immune indicator to evaluate the physical condition of cattle, wherein the higher the ADG of the cattle, the lower the immune response in the body and the better the health of the cattle. Dressing percentage is another indicator of economic benefit, with a higher dressing percentage indicating better growth performance [32,33]. The dressing percentage was similar between the Yunling and Simmental cattle in this work, whereas the data showed that the dressing percentage of the Wenshan cattle was lower (*p* < 0.05) than in the other tested breeds.

### 4.2. Chemical Composition Analysis

Beef is a source of protein that plays a vital role in the human diet [33,34]. Studies showed that a high-protein diet may reduce the weight and blood glucose of obese patients [35,36,37]. Wycherley et al. [37] suggested that a high-protein diet was beneficial in reducing the weight of type Ⅱ diabetes patients. From the perspective of health consumption, Yunling and Simmental cattle exhibit certain advantages compared to Wenshan cattle [36]. LD is a type of skeletal muscle, with collagen being the main structural protein of the skeletal muscle extracellular matrix [38], and the content of collagen exerting a great influence on the quality of meat. Our results showed no significant differences (*p* > 0.05) in collagen contents among the three breeds, indicating that collagen content was not the reason resulting in the differences in meat quality.

The distribution and fluidity of water in meat significantly impacted meat qualities such as tenderness, flavor, juiciness and appearance [39], among which tenderness, flavor and juiciness were the key factors when evaluating the palatability of the beef types [2]. The palatability of Simmental cattle was similar to that of Yunling cattle and lower than that of Wenshan cattle (*p* < 0.05). Intermuscular fat content was positively correlated with meat flavor, juiciness, and tenderness, and significantly improved muscle texture, firmness, and water retention [40,41]. Increasing numbers of people are paying attention to the fat content of beef [42]. In this study, the fat contents of Yunling and Simmental cattle were similar and showed greater improvement in muscle texture, firmness and water retention compared to Wenshan cattle.

According to the results of these tests, the differences in protein, collagen, moisture, and fat contents were only between species and not dependent on the aging process. There were no interactions between breed and aging time in regard to protein, collagen, moisture, and fat contents. These results were consistent with previous studies, wherein Li et al. [43] found that the moisture of Simmental × Luxi crossbred bulls beef did not show significant change (*p* > 0.05) with aging (day 0–11). Another study showed that the moisture and fat contents of Tajima Japanese black cattle beef exhibited no differences between days 4 and 7 [44].

### 4.3. pH and Water Loss Rate Analysis

Based on an earlier study [45], pH is an important criterion for meat quality. In this study, the pH values throughout 7 days of aging were in the range of 5.41–5.46, consistent with the development of anaerobic glycolysis [46]. The pH trend in our study showed a consistency with previous studies [47,48]. Over time, muscles use glycogen to generate energy, maintain some energy-wasting reactions, and produce lactic acid, leading to a drop in pH [48]. There was significant difference in pH observed between the different breeds on days 0 and 3 (*p* < 0.05). On day 3, a higher pH was observed in Simmental cattle beef versus Yunling and Wenshan cattle beef (*p* < 0.05). The significance of this interaction may be due to differences in glycogen consumption among the three breeds during aging. A study conducted by Marino et al. [47] also supported this view.

As the water loss rate increased, the ability of the muscle to retain its original moisture content decreased when acted upon by external forces, resulting in poorer muscle tenderness, more nutrients lost, and lower meat quality [49]. The three beef breeds responded differently on day 1 with respect to the water-loss rate. This interaction reflected the ability of the muscle to retain its original moisture, which was significantly different among the three breeds (*p* < 0.05).

### 4.4. Cooking Loss and Warner-Bratzler Shear Force Analysis

Cooking loss was related to the water loss during the cooking process of the meat, reflecting the determined water-related capacity levels of the meat samples at high temperatures, which showed a strong negative correlation with meat juiciness. For meat processing enterprises, high cooking loss not only reduces meat edibleness, but also affects the appearance of the meat, therefore, cooking loss represents an important economic indicator [50]. A breed × aging time interaction effect was found for cooking loss (*p* < 0.05), with Simmental beef showing the lowest water-loss rate after 7 days of aging, suggesting that Simmental beef decreased in juiciness the least after cooking on day 7.

WBSF also is considered to be an indicator of beef tenderness, and was mechanically evaluated based on the force necessary to cut muscle fibers [51]; WBSF values were negatively correlated with tenderness. The WBSF of the three beef breeds declined during the aging process, proving that meat tenderness was better with increased aging time (day 0–7). Our findings agreed with previous studies [20,48,52,53]. The tenderness of the Yunling and Wenshan beef was better than that of Simmental cattle on all test days (*p* < 0.05).

### 4.5. MFI and IMP Analysis

MFI is an indicator that reflects the integrity of the myofibrils and their skeleton proteins in myocytes. A previous study showed that MFI was positively correlated with meat tenderness [54]. The MFI of the three breeds significantly rose at days 0, 3 and 7 (*p* < 0.05). These results were consistent with previous published research [52].

IMP, as well as its degradation product, hypoxanthine, were deemed to be vital constituents in the intensity of meat [44]. Over time, hypoxanthine contents were shown to increase in pork, alongside a simultaneous decrease in the concentration of IMP and increasing bitterness and saltiness intensity [27]. The rates of decreasing IMP contents in Yunling and Wenshan cattle were lower than in Simmental cattle, thereby confirming that the increased rates of bitterness and saltiness in Yunling and Wenshan beef were lower than in Simmental cattle (*p* < 0.05).

### 4.6. Color Measurement Analysis

Meat color is an important determinant of visual appearance, and also reflects meat quality and freshness. Meat color is determined by the presence of three chemical forms of myoglobin, namely, reducing myoglobin (amaranth), oxymyoglobin (bright red), and metmyoglobin (brown). Over time, as beef oxidizes, beef color changes from purple to bright red and, finally, to brown [55,56,57,58]. Oxymyoglobin content was the main cause of changes in a* value, and was shown to be positively correlated with a* value [59]; in other words, the beef samples exhibited larger a* values as time increased, leading to a more intense redness, consistent with the results of Marino et al. [47]. The beef samples of the three breeds were all observed to be brighter and yellower as the L* value increased on day 0 compared with day 7. Overall, beef color generally became brighter and showed more intense red and yellow colors during the aging process.

### 4.7. Fatty Acid Profile Analysis

Fatty acids are often classified as saturated fatty acids (SFA), monounsaturated fatty acids (MUFA) and polyunsaturated fatty acids (PUFA) [60]. The percentages of fatty acids from each beef sample showed negligible variation during the aging process. Regarding the phenomenon that the beef fatty acid profiles were unchanged after 7 days of aging (*p* > 0.05), a recent study put forward that natural antioxidants in meat play important roles during the first 7 days postmortem [61]. The individual fatty acid percentages were different between the three breeds on certain aging days (*p* < 0.05), with SFA contents observed to be higher in Yunling beef than in Simmental and Wenshan beef at days 0, 3 and 7 (*p* < 0.05). In contrast, MUFA contents were lower in Yunling beef than in Simmental and Wenshan beef at days 0, 3 and 7 (*p* < 0.05). Reducing dietary SFA, increasing MUFA, or decreasing PUFA (n−6/n−3) contents in meat may decrease the risk of cardiovascular disease [62,63]. Further, the SFAs in the Yunling beef had the potential to reduce quality improvement.

According to reports by Cameron et al. and Wood et al., the nutritional value of meat can be measured according to the ratio of PUFAs to SFAs (P:S) [41,64]. The World Health Organization (WHO) recommends a P:S ratio in the diet of higher than 0.4 [65]. Due to ruminal hydrogenation, the contents of unsaturated fatty acids (UFAs) in beef, especially PUFAs, were lower than in monogastric animals, with a P:S value in beef of about 0.1 [66]. The P:S values of the three beef breeds in this work were all higher than 0.1. Based on present data, from a P:S point of view, these three breeds of beef could be considered to meet the recommended health requirements of consumers.

Studies showed that elevated cholesterol levels are detrimental to health [67]. Studies also found that dietary C16:0 could lower serum cholesterol levels [68]. Another fatty acid considered to be good for cardiovascular health is C18:2n6c, an essential fatty acid [69]. The C18:2n6c content of Yunling cattle was significantly higher than that of Simmental cattle and Wenshan cattle (*p* < 0.05). Thus, ingestion of Yunling beef may be beneficial to facilitate improvement of cholesterol levels compared to ingestion of Simmental and Wenshan beef.

## 5. Conclusions

The data obtained in this study prove that Yunling cattle are basically no different in growth performance, carcass traits, pH, and water-holding capacity than Simmental cattle. The fatty acid profiles of Yunling beef observed here were conducive with improved cholesterol levels and almost unaffected by aging time. The tenderness and meat color became better in Yunling beef as the aging time increased, implying that Yunling beef aged for 7 days was more suitable in regard to cooking and sensory characteristics. Therefore, a 7-day, short-term aging process is very effective in improving the quality of Yunling beef.

## Figures and Tables

**Table 1:** The growth performance and carcass trait of the cattle breeds.

Item	Yunling Cattle	Simmental Cattle	Wenshan Cattle	SEM	Effects, *p*
Initial weight (kg)	305 ^a^	312 ^a^	160 ^b^	3.45	<0.0001
Final weight (kg)	498 ^b^	540 ^a^	326 ^c^	2.70	<0.0001
Body weight (kg)	487 ^b^	526 ^a^	312 ^c^	3.19	<0.0001
Average daily gain (kg/day)	1.07 ^ab^	1.26 ^a^	0.92 ^b^	0.04	0.0021
Carcass weight (kg)	295 ^a^	318 ^a^	178 ^b^	2.61	<0.0001
Dressing percentage (%)	61.46 ^a^	60.24 ^a^	57.49 ^b^	0.31	0.0325

Note: ^a,b,c^ = *p* < 0.05 in the row (breed effect).

**Table 2:** The chemical composition of Yunling, Simmental and Wenshan beef during aging.

Item	Day	Breed			SEM	Effects, *p*		
		Yunling Cattle	Simmental Cattle	Wenshan Cattle		Breed	Aging	Breed × Aging
Protein (%)	0	23.55 ^b^	26.04 ^a^	23.18 ^b^	0.13	0.0316	0.9523	0.4128
	3	23.49 ^b^	26.08 ^a^	23.12 ^b^				
	7	23.47 ^b^	26.01 ^a^	23.15 ^b^				
Collagen (%)	0	0.71	0.73	0.75	0.02	0.9075	0.9157	0.1137
	3	0.69	0.71	0.72				
	7	0.72	0.74	0.74				
Moisture (%)	0	72.51 ^b^	72.24 ^b^	74.78 ^a^	0.56	0.0452	0.9256	0.6024
	3	72.63 ^b^	72.15 ^b^	74.86 ^a^				
	7	72.42 ^b^	71.94 ^b^	74.98 ^a^				
Fat (%)	0	3.47 ^a^	3.57 ^a^	3.05 ^b^	0.08	0.0012	0.9416	0.0896
	3	3.41 ^a^	3.50 ^a^	3.01 ^b^				
	7	3.39 ^a^	3.61 ^a^	3.06 ^b^				

Note: ^a,b,c^ = *p* < 0.05 in the row (breed effect).

**Table 3:** The pH and water loss rate affected by breed and aging.

Item	Day	Breed			SEM	Effects, *p*		
		Yunling Cattle	Simmental Cattle	Wenshan Cattle		Breed	Aging	Breed × Aging
pH	0	6.23 ^Aab^	6.10 ^Ab^	6.39 ^Aa^	0.06	0.1227	<0.0001	0.0005
	1	5.52 ^B^	5.53 ^B^	5.50 ^B^				
	2	5.45 ^BC^	5.39 ^C^	5.44 ^C^				
	3	5.35 ^Cb^	5.46 ^BCa^	5.37 ^BCb^				
	5	5.51 ^B^	5.43 ^BC^	5.46 ^BC^				
	7	5.46 ^BC^	5.41 ^BC^	5.43 ^BC^				
Water loss rate (%)	0	23.05	22.61 ^BC^	21.42 ^B^	0.37	0.1367	0.0003	0.0105
	1	23.36 ^a^	18.83 ^Db^	24.32 ^Aa^				
	2	24.21	21.89 ^CD^	23.99 ^A^				
	3	23.49	25.83 ^AB^	24.37 ^A^				
	5	24.14	23.80 ^ABC^	25.72 ^A^				
	7	23.90	26.10 ^A^	26.30 ^A^				

Note: ^A,B,C^ = *p* < 0.05 in the column (aging effect). ^a,b,c^ = *p* < 0.05 in the row (breed effect).

**Table 4:** The cooking loss and Warner-Bratzler shear force (WBSF) affected by breed and aging.

Item	Day	Breed			SEM	Effects, *p*		
		Yunling Cattle	Simmental Cattle	Wenshan Cattle		Breed	Aging	Breed × Aging
Cooking loss (%)	0	28.42 ^Ba^	21.87 ^Db^	26.97 ^Ba^	0.32	<0.0001	<0.0001	<0.0001
	1	29.26 ^Ba^	25.54 ^Cb^	29.91 ^Aa^				
	2	26.55 ^Cb^	30.62 ^Aa^	31.33 ^Aa^				
	3	32.50 ^Aa^	28.69 ^ABb^	31.90 ^Aa^				
	5	30.14 ^B^	29.47 ^AB^	31.31 ^A^				
	7	32.21 ^Aa^	28.22 ^Bb^	31.61 ^Aa^				
WBSF (N)	0	86.71 ^A^^a^	73.45 ^A^^b^	90.17 ^A^^a^	1.26	0.0125	<0.0001	0.0068
	1	86.46 ^A^^a^	71.64 ^AB^^b^	86.52 ^ABa^				
	2	84.54 ^AB^^a^	69.03 ^AB^^b^	86.40 ^AB^^a^				
	3	79.65 ^B^^a^	65.47 ^B^^b^	78.58 ^B^^a^				
	5	71.61 ^C^^a^	64.95 ^B^^b^	73.55 ^C^^a^				
	7	65.32 ^D^^a^	60.07 ^C^^b^	67.58 ^D^^a^				

Note: ^A,B,C^ = *p* < 0.05 in the column (aging effect). ^a,b,c^ = *p* < 0.05 in the row (breed effect).

**Table 5:** The MFI and IMP affected by breed and aging.

Item	Day	Breed			SEM	Effects, *p*		
		Yunling Cattle	Simmental Cattle	Wenshan Cattle		Breed	Aging	Breed × Aging
MFI (mg/mL)	0	97.56 ^Ca^	61.15 ^Cb^	62.12 ^Cb^	2.01	<0.0001	<0.0001	0.1225
	3	120.40 ^Ba^	73.80 ^Bb^	76.18 ^Bb^				
	7	138.77 ^Aa^	96.70 ^Ab^	95.65 ^Ab^				
IMP (μg/g)	0	896.11 ^Aa^	805.98 ^Ab^	822.17 ^Ab^	8.26	<0.0001	<0.0001	0.8319
	3	764.71 ^Ba^	558.28 ^Bb^	806.26 ^Aa^				
	7	541.41 ^C^	532.58 ^B^	546.38 ^B^				

Note: ^A,B,C^ = *p* < 0.05 in the column (aging effect). ^a,b,c^ = *p* < 0.05 in the row (breed effect).

**Table 6:** Muscle color parameters measured at different aging days.

Item	Day	Breed			SEM	Effects, *p*		
		Yunling Cattle	Simmental Cattle	Wenshan Cattle		Breed	Aging	Breed × Aging
L*	0	28.20 ^Ca^	25.64 ^Cb^	27.33 ^Da^	0.47	<0.0001	<0.0001	0.8956
	1	31.15 ^BC^	30.31 ^B^	33.87 ^BC^				
	2	33.66 ^AB^	33.10 ^AB^	33.47 ^C^				
	3	35.81 ^A^	34.53 ^A^	35.36 ^AB^				
	5	35.63 ^A^	33.79 ^A^	35.68 ^A^				
	7	36.15 ^A^	34.01 ^A^	35.99 ^A^				
a*	0	11.63 ^Bab^	12.31 ^Ca^	11.25 ^b^	0.23	<0.0001	<0.0001	0.4195
	1	16.56 ^A^	16.70 ^B^	15.69 ^A^				
	2	16.53 ^Aab^	18.86 ^Aa^	16.00 ^Ab^				
	3	16.64 ^A^	18.60 ^A^	16.68 ^A^				
	5	16.26 ^Aab^	16.58 ^Ba^	15.10 ^Ab^				
	7	16.45 ^Aab^	17.66 ^ABa^	15.67 ^Ab^				
b*	0	0.99 ^B^	1.16 ^C^	1.02 ^C^	0.17	0.2641	<0.0001	0.4333
	1	5.00 ^A^	4.39 ^B^	4.55 ^AB^				
	2	4.83 ^A^	6.37 ^A^	4.91 ^AB^				
	3	5.28 ^A^	6.33 ^A^	5.81 ^A^				
	5	5.40 ^A^	4.75 ^B^	4.14 ^B^				
	7	4.86 ^A^	5.15 ^B^	4.72 ^AB^				

Note: ^A,B,C^ = *p* < 0.05 in the column (aging effect). ^a,b,c^ = *p* < 0.05 in the row (breed effect).

**Table 7:** The saturated fatty acids content affected by breed and aging.

Fatty Acid	Day	Breed			SEM	Effects, *p*		
		Yunling Cattle	Simmental Cattle	Wenshan Cattle		Breed	Aging	Breed × Aging
C4:0 (%)	0	0.31 ^a^	0.18 ^c^	0.23 ^b^	0.01	<0.0001	0.8232	0.1238
	3	0.29 ^a^	0.19 ^c^	0.22 ^b^				
	7	0.32 ^a^	0.19 ^c^	0.23 ^b^				
C10:0 (%)	0	0.01 ^b^	0.03 ^a^	0.03 ^a^	0.00	<0.0001	0.9975	0.3791
	3	0.01 ^b^	0.03 ^a^	0.03 ^a^				
	7	0.01 ^b^	0.03 ^a^	0.03 ^a^				
C12:0 (%)	0	0.01 ^c^	0.02 ^b^	0.04 ^a^	0.00	<0.0001	0.9888	0.6059
	3	0.01 ^c^	0.02 ^b^	0.04 ^a^				
	7	0.01 ^c^	0.02 ^b^	0.04 ^a^				
C14:0 (%)	0	2.37 ^b^	2.39 ^b^	3.21 ^a^	0.03	<0.0001	0.8677	0.6642
	3	2.40 ^b^	2.37 ^b^	3.28 ^a^				
	7	2.41 ^b^	2.41 ^b^	3.25 ^a^				
C15:0 (%)	0	0.17 ^c^	0.31 ^a^	0.22 ^b^	0.01	<0.0001	0.8291	0.7785
	3	0.18 ^c^	0.34 ^a^	0.20 ^b^				
	7	0.17 ^c^	0.33 ^a^	0.21 ^b^				
C16:0 (%)	0	29.21 ^a^	27.83 ^b^	27.61 ^b^	0.52	0.0127	0.8327	0.4725
	3	29.19 ^a^	28.01 ^b^	27.69 ^b^				
	7	29.17 ^a^	27.83 ^b^	27.71 ^b^				
C17:0 (%)	0	0.52	0.49	0.50	0.01	0.8987	0.7537	0.4126
	3	0.54	0.52	0.52				
	7	0.52	0.51	0.48				
C18:0 (%)	0	15.26 ^a^	14.37 ^b^	13.32 ^c^	0.37	0.0112	0.9918	0.8459
	3	15.30 ^a^	14.33 ^b^	13.34 ^c^				
	7	15.46 ^a^	14.34 ^b^	13.29 ^c^				
C22:0 (%)	0	0.23 ^a^	0.06 ^b^	0.05 ^b^	0.01	<0.0001	0.8901	0.8754
	3	0.24 ^a^	0.07 ^b^	0.05 ^b^				
	7	0.23 ^a^	0.07 ^b^	0.05 ^b^				
ΣSFA (%)	0	48.14 ^a^	45.68 ^b^	45.61 ^b^	0.98	0.0028	0.8903	0.6894
	3	48.16 ^a^	45.88 ^b^	45.86 ^b^				
	7	48.34 ^a^	45.73 ^b^	45.74 ^b^				

Note: ^A,B,C^ = *p* < 0.05 in the column (aging effect). ^a,b,c^ = *p* < 0.05 in the row (breed effect).

**Table 8:** The monounsaturated fatty acids content affected by breed and aging.

Fatty Acid	Day	Breed			SEM	Effects, *p*		
		Yunling Cattle	Simmental Cattle	Wenshan Cattle		Breed	Aging	Breed × Aging
C14:1 (%)	0	0.64 ^b^	0.56 ^b^	0.92 ^a^	0.01	<0.0001	0.8835	0.7684
	3	0.62 ^b^	0.54 ^b^	0.87 ^a^				
	7	0.61 ^b^	0.57 ^b^	1.01 a				
C15:1 (%)	0	0.05 ^b^	0.07 ^a^	0.05 ^b^	0.01	0.0015	0.9612	0.0926
	3	0.05 ^b^	0.08 ^a^	0.05 ^b^				
	7	0.04 ^b^	0.08 ^a^	0.05 ^b^				
C16:1 (%)	0	3.01 ^c^	3.59 ^b^	4.15 ^a^	0.03	<0.0001	0.8262	0.8126
	3	2.96 ^c^	3.64 ^b^	4.12 ^a^				
	7	2.94 ^c^	3.61 ^b^	4.10 ^a^				
C17:1 (%)	0	0.32 ^b^	0.39 ^ab^	0.43 ^a^	0.01	0.0024	0.8609	0.6618
	3	0.34 ^b^	0.39 ^ab^	0.40 ^a^				
	7	0.33 ^b^	0.38 ^ab^	0.41 ^a^				
C18:1n9c (%)	0	39.65 ^b^	42.61 ^a^	41.90 ^ab^	1.17	0.0013	0.8966	0.8269
	3	39.72 ^b^	42.49 ^a^	41.81 ^ab^				
	7	39.62 ^b^	42.57 ^a^	41.85 ^ab^				
C18:1n9t (%)	0	0.49 ^a^	0.30 ^b^	0.46 ^a^	0.01	<0.0001	0.8353	0.1265
	3	0.47 ^a^	0.29 ^b^	0.44 ^a^				
	7	0.45 ^a^	0.29 ^b^	0.43 ^a^				
C20:1 (%)	0	0.34 ^b^	0.31 ^b^	0.41 ^a^	0.01	0.0089	0.8735	0.1026
	3	0.31 ^b^	0.34 ^b^	0.42 ^a^				
	7	0.34 ^b^	0.32 ^b^	0.42 ^a^				
C22:1n9 (%)	0	1.32 ^a^	1.05 ^b^	0.83 ^c^	0.02	<0.0001	0.8195	0.5496
	3	1.35 ^a^	0.98 ^b^	0.86 ^c^				
	7	1.37 ^a^	0.96 ^b^	0.82 ^c^				
Σ MUFA (%)	0	45.86 ^b^	48.86 ^a^	49.20 ^a^	1.05	0.0032	0.8303	0.2195
	3	45.82 ^b^	48.69 ^a^	48.97 ^a^				
	7	45.70 ^b^	48.78 ^a^	49.09 ^a^				

Note: ^A,B,C^ = *p* < 0.05 in the column (aging effect). ^a,b,c^ = *p* < 0.05 in the row (breed effect).

**Table 9:** The polyunsaturated fatty acids content affected by breed and aging.

Fatty Acid	Day	Breed			SEM	Effects, *p*		
		Yunling Cattle	Simmental Cattle	Wenshan Cattle		Breed	Aging	Breed × Aging
C18:2n6c (%)	0	5.18 ^a^	4.70 ^ab^	4.36 ^b^	0.08	<0.0001	0.9103	0.8529
	3	5.21 ^a^	4.63 ^ab^	4.33 ^b^				
	7	5.17 ^a^	4.68 ^ab^	4.35 ^b^				
C18:2n6t (%)	0	0.52	0.50	0.51	0.02	0.9055	0.8646	0.1237
	3	0.54	0.52	0.53				
	7	0.51	0.53	0.52				
C18:3n3 (%)	0	0.25	0.22	0.27	0.01	0.8218	0.8194	0.0988
	3	0.22	0.24	0.27				
	7	0.23	0.23	0.25				
C22:6n3 (%)	0	0.05	0.04	0.05	0.01	0.9137	0.9320	0.0784
	3	0.05	0.04	0.04				
	7	0.05	0.05	0.05				
ΣPUFA (%)	0	6.00 ^a^	5.46 ^ab^	5.19 ^b^	0.07	<0.0001	0.8508	0.8741
	3	6.02 ^a^	5.43 ^ab^	5.17 ^b^				
	7	5.96 ^a^	5.49 ^ab^	5.17 ^b^				
ΣPUFA/ΣSFA (P:S)	0	0.12	0.12	0.11	0.01	0.8356	0.9806	0.1128
	3	0.13	0.12	0.11				
	7	0.12	0.12	0.11				

Note: ^A,B,C^ = *p* < 0.05 in the column (aging effect). ^a,b,c^ = *p* < 0.05 in the row (breed effect).

## Supplementary Materials

The following are available online at https://www.mdpi.com/2076-2615/10/10/1897/s1. Table S1: Composition and nutrient levels.
