# Supplementary material for: Effect of Aging Time on Meat Quality of Longissimus Dorsi from Yunling Cattle: A New Hybrid Beef Cattle
Source: Animals (Basel). 2020 Oct 16;10(10):1897. doi: 10.3390/ani10101897 (PMC7602736; doi:10.3390/ani10101897)
Supplement: Supplementary file 1 [file animals-10-01897-s001.pdf]

animals

Table S1. Composition and nutrient levels.

Ingredients	Diet (%)	Nutrient levels	Diet
Corn	62.00	NE(MJ/kg)	7.94
Soybean meal	12.00	CP	12.97
Cottonseed meal	8.00	Ca	0.60
$CaHPO_4$	1.50	P	0.64
NaCl	0.30		
Premix	0.70		
Barley	22.00		
Fat	1.00		
Soda	0.50		

NE: net energy.
